# The Role of Square Dancing in Psychological Capital: Evidence from a Large Cross-Sequential Study

**DOI:** 10.3390/healthcare13151913

**Published:** 2025-08-05

**Authors:** Ruitong Li, Yujia Qu, Zhiyuan Liu, Yan Wang

**Affiliations:** 1 Department of Education, Beijing Sport University, Beijing 100084, China; liruitong@bsu.edu.cn; 2 Department of Health and Physical Education, The Education University of Hong Kong, Tai Po, New Territories, Hong Kong SAR, China; qyjnnn15940302640@163.com; 3 Faculty of Psychology, Beijing Normal University, Beijing 100875, China; 202421061032@mail.bnu.edu.cn

**Keywords:** square-dancing, cross-sequential study, psychological capital (PsyCap), middle-aged and older adults, cognitive reappraisal, prosocial behavior tendency

## Abstract

(1) **Background**: Rapid population aging in China intensifies physical and mental health challenges, including negative emotions and social barriers. Physical activity (PA) fosters resilience, adaptability, and successful aging through emotional and social benefits. This study examines the relationship between square-dancing exercise and psychological capital (PsyCap) in middle-aged and elderly individuals using cross-validation, subgroup analysis, and a cross-sequential design. (2) **Methods**: A cross-sectional study with 5714 participants employed a serial mediation model. Online questionnaires assessed square-dancing exercise, cognitive reappraisal, prosocial behavior tendencies, PsyCap, and interpersonal relationships. Statistical analyses were conducted using SPSS 27.0 and Mplus 8.3, incorporating correlation analysis, structural equation modeling, and subgroup comparisons. (3) **Results**: (a) Cognitive reappraisal and prosocial behavior mediated the link between square-dancing and PsyCap through three pathways; (b) model stability was confirmed across two random subsamples; (c) cross-group differences emerged in age and interpersonal relationships. Compared with secondary data, this study further validated PsyCap’s stability over six months post-pandemic. (4) **Conclusions**: The study, based on China’s largest square-dancing sample, establishes a robust serial mediation model. The findings strengthen theoretical foundations for PA-based interventions promoting psychological resilience in aging populations, highlighting structured exercise’s role in mental and social well-being.

## 1. Introduction

Population aging has become a global issue affecting human development, and the social problems of old age and health caused by aging are becoming increasingly prominent [1]. China will become one of the countries with the highest proportion of elderly people in the world, and the scale, depth, and speed of population aging will inevitably lead to a series of physical and mental health problems [2], such as negative emotions, social barriers, and psychosocial and environmental factors [3]. To address these challenges, increasing attention has been given to the role of psychological capital (PsyCap), a positive psychological resource that can help middle-aged and older adults better cope with the questions brought about by aging [4].

PsyCap, a positive psychological resource, has a cumulative effect on the mental health of middle-aged and older adults [5]. Recent research highlights that PsyCap in older adults is significantly associated with enhanced mental health and positive emotional experiences [4] and further contributes to successful aging by improving adaptability [6]. Physical activity (PA) serves as a vital intervention for aging populations, offering multifaceted benefits in disease prevention, health promotion, and the cultivation of positive psychological resources [7]. A 20-year prospective cohort study highlighted the profound impact of PA on life expectancy among older adults [8], underscoring its role in fostering resilience and well-being. According to the broaden-and-build theory of positive emotions [9], middle-aged and older adults are able to cope with the challenges of retirement transition, health decline, and social role change by developing greater emotion regulation and effectively mobilizing external resources to generate high PsyCap through participation in PA [10] and volunteer experiences [11].

PA can be undertaken in a variety of ways, such as individual activities (e.g., walking, cycling, etc.) and group activities (e.g., square-dancing, aerobics dance, etc.). Among various forms of physical activity, owing to their unique social, recreational, and fitness-oriented characteristics, square dancing has gained immense popularity among middle-aged and older adults in China [12]. Square dancing, as a unique form of PA, amplifies these benefits through its dual-path mechanism: (1) its rhythmic, music-driven choreography facilitates cognitive reappraisal, enabling participants to reframe stressors (e.g., aging anxieties) into manageable response strategies [13]; (2) its group-based format fosters prosocial behaviors through collective coordination and trust-building [14]. These pathways synergistically increase PsyCap: cognitive reappraisal strengthens internal psychological resources, whereas prosocial behaviors expand external social networks, enabling individuals to reconstruct meaning in life, set adaptive goals, and sustain well-being.

### 1.1. Square-Dancing and PsyCap

PsyCap, a positive psychological construct formed during an individual’s growth and development, encompasses four core elements—self-efficacy, optimism, hope, and resilience [15]—and serves as a measurable and developable predictor of attitudes, behaviors, and performance. PsyCap is a psychological resource that can be increased in a variety of ways (e.g., through social relationships and group activities), enables individuals to feel positive psychological qualities and energy, such as hope and self-confidence, stimulates positive emotions, and encourages individuals to actively participate in social activities [16]. Recent research specifically highlights group-based exercises such as square dancing as potent interventions for PsyCap development through collective goal pursuit and mutual support [17]. In addition, in group programs, group members engage in regular physical exercise for a long period, often teaching and learning from each other, increasing their skill levels each day, and increasing self-confidence, hope, optimism, and resilience [18]. In summary, we propose H1: square-dancing exercise positively predicts PsyCap in middle-aged and older adults.

### 1.2. Relationships Between Cognitive Reappraisal and PsyCap

Cognitive reappraisal is an antecedent-focused emotion regulation strategy that alters emotional responses by modifying the interpretation of emotionally salient situations [19] and is related to a series of developmental factors, such as social interaction, social adaptation, mental health, and a range of other developmental outcomes [20]. In recent years, there has been a gradual shift in the study of emotions from the individual perspective alone to a social perspective. For example, interpersonal interactions improve middle-aged and older adults’ self-control over their emotions [21].

Rhythmic aerobic exercise is an important method to promote emotion regulation and improve cognitive reappraisal ability. Individuals’ ability to fully utilize cognitive reappraisal strategies during PA can help reduce their negative emotional experiences and produce more positive emotional states [22]. Regular participation in square-dancing exercise enables middle-aged and older adults to form major social interaction bonds and is an important way to influence their mood. In summary, we propose H2: cognitive reappraisal mediates the relationship between square-dancing exercise and PsyCap.

### 1.3. Relationships Between Prosocial Behavior Tendencies and PsyCap

Prosocial behaviors include all behaviors that meet social expectations and are beneficial to groups and society, mainly in the three aspects of sharing, cooperating, and helping others [23]. Social cognitive theory suggests that people tend to address social problems by considering the perceptions of others and focusing on their reactions as a way of adapting to the current social environment [24].

Members who have often helped others are prone to positive emotional experiences, while face-to-face interaction activities between dancers generate new friendships, effectively generating mutual assistance behaviors and increasing the level of well-being experience among members [25]. There is a correlation between PA and prosocial behavioral intentions in middle-aged and older adults, i.e., the amount of PA of an individual positively predicts his or her prosocial behavioral intentions [26]. In the process of square-dancing exercise, people with the same exercise goal can increase their emotional connection with each other, which effectively promotes improvements in helping and reciprocity behaviors, generates a stronger tendency toward prosocial behaviors, and further increases their PsyCap. In summary, we propose H3: prosocial behavior tendencies mediate the relationship between square-dancing exercise and PsyCap.

### 1.4. Serial Multiple Mediation Mechanism of the Relationship Between Cognitive Reappraisal and Prosocial Behavior

Socioemotional selectivity theory (SSI) suggests that with age, middle-aged and older adults gradually realize that life is limited, so they compensate for the loss of internal and external resources by optimizing the process of emotion regulation [27]. Cognitive reappraisal is an effective means of reducing negative emotions and increasing an individual’s tendency toward prosocial behavior. Individuals who use cognitive reappraisal strategies to view a problem are highly sensitive to situational information, can have good social interactions, and are more likely to engage in prosocial behavior [28].

Moreover, individuals who regularly use cognitive reappraisal strategies tend to be more satisfied with and optimistic about their lives, show greater autonomy, have more positive interpersonal relationships, and produce stronger predispositions to prosocial behavior. Square-dancing exercise helps increase individuals’ positive emotions, meets middle-aged and older adults’ needs for social interaction and emotional exchange [29], and effectively increases social participation and prosocial behavior, which in turn increases their PsyCap. Therefore, we propose H4: cognitive reappraisal and prosocial behavior tendencies have a serial mediating effect on the association between square-dancing exercise and PsyCap.

### 1.5. Differences in Intermediary Roles

Based on previous studies, we found that demographic variables and interpersonal interactions affect the cultivation and development of psychological resources. From a demographic perspective, age and socioeconomic status (e.g., income level) are key factors influencing an individual’s level of PsyCap [30]. In terms of age, compared with middle-aged adults, older adults use more cognitive reappraisal strategies, have more pronounced means of prosocial behavioral performance, and have higher levels of mental toughness [31]. In terms of income, there is a significant difference in the mechanisms of PsyCap formation between low-income and high-income individuals [32]. Accordingly, the present exploratory study further investigated possible age and income differences in chain mediation.

From the perspective of social interactions, interpersonal distress is a contradictory or conflicting psychological state formed by individuals in interpersonal interactions. Elderly people, owing to differences in their personal views and degree of participation in social interactions, are prone to interpersonal distress, which may result in negative psychological tendencies, i.e., social isolation, loneliness, etc. [33]. Therefore, this study takes the perspective of the level of interpersonal distress to explore whether interpersonal relationship variables can influence changes in the chain mediation model to further clarify the need to decrease the level of interpersonal distress. In summary, three differences in the mediating role are proposed: H5: Cognitive reappraisal and prosocial behavior mediate the relationships among square-dancing exercise, PsyCap, monthly income, age, and degree of interpersonal relationship distress.

### 1.6. The Present Study

In recent years, most studies have explored the concepts and theoretical foundations of PsyCap and its structure and measurement, primarily within the domain of organizational behavior, with a predominant focus on employee populations [34]. Research has shown that PsyCap positively influences job satisfaction, performance, and organizational commitment [35]. However, a notable gap remains in the literature regarding PsyCap’s impact on diverse populations such as adolescents and older adults, particularly in nonorganizational contexts like community engagement and health promotion. Current research faces several limitations: (1) Although PsyCap benefits athletic performance, mental health, and stress resilience among student-athletes and adolescents [36], studies on adolescents in non-sport settings and older adults are scarce. PsyCap has been linked to youth mental health in schools [37] and well-being among rural seniors [38], highlighting the need for broader investigations beyond workplace-centric paradigms and considering the diverse applications of PsyCap across the lifespan and in varied sociocultural environments. (2) Many PsyCap studies rely on small samples, correlational designs, and null hypothesis testing, limiting generalizability and increasing false positives. Some lack transparency and robust measurement validation [39].

**Figure 1:**
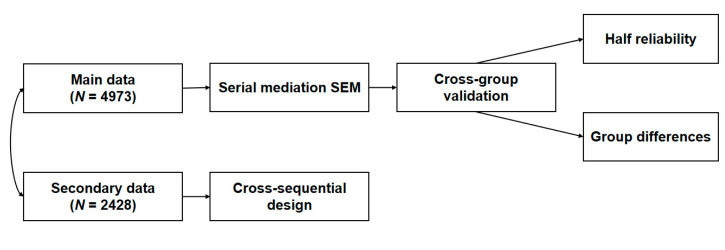
Flowchart of the present study design. Notes: The secondary data used in this study were obtained from a previous study [40].

To our knowledge, this is the first study that uses the randomized split-half strategy among a large sample while further examining the uniformity of the subgroup analysis in the Chinese context. Therefore, the purpose of this study was to explore the relationships between square-dancing exercise and PsyCap and the intrinsic mechanism from a positive psychology perspective by combining a cross-sequential design and cross-validation. The specific process is shown in Figure 1.

## 2. Materials and Methods

### 2.1. Data Sources

#### 2.1.1. Main Data

A total of 5714 square-dancing participants were recruited online by Wenjuanxing (https://www.wjx.cn/ (accessed on 12 September 2023).) from 19 August to 12 September 2023 in China. We monitored the IP addresses of the respondents to avoid multiple responses. The questionnaire was distributed across 30 provinces, autonomous regions, and municipalities, resulting in a wide geographical distribution of the sample, including Zhejiang, Beijing, Jiangsu, Guangdong, Fujian, Inner Mongolia, Shanghai, Qinghai, Shandong, Yunnan, Hunan, and Chongqing.

To ensure the accuracy of the data, on the basis of the measurement experience [41], we analyzed the data according to the response time of middle-aged and older adults, as well as the total number of questions, and eliminated those submitted too quickly (the average time spent on each question was less than 2 s), those with unanswered questions (omission of the questions), those from respondents who did not meet the age requirement, and those from respondents who failed the attention check. Our final sample consisted of 4973 individuals, 95.7% of whom were women (*M*_age_ =59.14 ± 7.17 years).

#### 2.1.2. Secondary Data

The data were collected by Qu et al. (2023) [40], who investigated the demographic characteristics, PAs, and other psychological variables of middle-aged and older adults aged 45 years between March and April 2023 after the restrictions loosened. Our secondary sample consisted of 2428 individuals, 95.2% of whom were female (*M*_age_ = 60.62 ± 6.68 years).

This dataset, which uses the same measurement instruments as the main data used in this study and has more consistent characteristics with the target population, will be used as a cross-sequential design and compared with a previous study [40] to demonstrate and examine the robustness of the PsyCap of middle-aged and older square dance groups over almost 6 months post-pandemic.

#### 2.1.3. Cross-Sequential Design

The cross-sequential design is a robust method for examining developmental patterns, combining the strengths of both cross-sectional and longitudinal approaches [42]. It is particularly useful for assessing the temporal stability and generalizability of psychological constructs in specific populations and contexts, such as post-pandemic recovery [43].

In this study, we compared two independent samples of middle-aged and older square-dancing participants, collected approximately six months apart, using identical measurement instruments and targeting similar demographic groups. This design enabled us to examine the stability and generalizability of PsyCap and PA during a six-month post-pandemic recovery period. By leveraging the cross-sequential approach, our research offers insights into how PsyCap evolves over time and across cohorts, highlighting its potential as a stable psychological resource in middle-aged and older populations.

### 2.2. Measures

#### 2.2.1. Demographic Information

Participants provided demographic details through a structured questionnaire, including sex, age, monthly income, marital status, square-dancing intensity and duration, typical group size, and interpersonal relationships within the exercise context.

#### 2.2.2. Square-Dancing Exercise

Square-dancing was measured using the Physical Activity Rating Scale-3 (PARS-3), originally developed by Liang [44]. This scale evaluates square-dancing over the past month through three components: intensity, duration, and frequency. The total activity score is calculated as exercise amount = intensity × duration × frequency, with intensity and frequency rated on a 1–5 scale and duration on a 0–4 scale. Scores range from 0 to 100, with higher scores indicating a greater level of square-dancing. The internal consistency (Cronbach’s α) was 0.65, and the test–retest reliability for individual items reached 0.83.

#### 2.2.3. Psychological Capital

PsyCap was assessed using the Positive Psychological Questionnaire (PPQ), adapted from Zhang [45]. The PPQ captures four core dimensions of PsyCap: self-efficacy, hope, optimism, and resilience [30]. Items are rated on a 7-point Likert scale, and mean scores are computed, with higher values reflecting stronger PsyCap. In this study, the scale demonstrated high internal reliability (Cronbach’s α = 0.91).

#### 2.2.4. Cognitive Reappraisal

Cognitive reappraisal was assessed using the Emotion Regulation Questionnaire (ERQ) to examine individuals’ choices of cognitive reappraisal and expressive inhibition to cope with emotional reactions [19]. In this study, the Chinese version of the ERQ was used (Wang) [46]. The questionnaire contains two dimensions with ten items each: cognitive reappraisal and expression suppression. It is scored on a 7-point scale. The items are averaged, with higher mean scores indicating that the participant utilized the emotion regulation strategy more frequently. In this study, Cronbach’s α was 0.90.

#### 2.2.5. Prosocial Behavior Tendency

Prosocial behavior tendency was assessed using the Prosocial Tendencies Measure (PTM) to examine various prosocial tendencies [47]. In this study, the Chinese version of the PTM was used (Kou) [48]. The questionnaire contains six dimensions with twenty-six items each: emotional, obedient, altruistic, anonymous, public, and urgent. It is scored on a 5-point scale. The items are averaged, with higher mean scores indicating more prosocial behavior tendencies. In this study, Cronbach’s α was 0.95.

#### 2.2.6. Interpersonal Relationships

Interpersonal relationships were assessed using the Interpersonal Comprehensive Diagnostic Scale (ICDS) to assess subjects’ perceived interpersonal distress [49]. The questionnaire contains four dimensions with twenty-eight items each: “conversation and communication”, “socializing and friendship”, “the way one treats people”, and “heterosexual relationships”. It is scored on a 2-point scale. The items are averaged, with higher mean scores indicating more severe interpersonal behavioral distress. In this study, Cronbach’s α was 0.92.

### 2.3. Statistical Analysis

The statistical analysis of the data was carried out using IBM SPSS Statistics for Windows, Version 27.0 (SPSS Inc., IBM Company, Armonk, NY, USA) and Mplus, Version 8.3 (Muthén & Muthén, Los Angeles, CA, USA), with a significance level set at 0.05. SPSS 27.0 was used to calculate and describe demographic characteristics, as well as to examine variable correlations using t-tests. Prior to conducting structural equation modeling (SEM), multicollinearity among observed variables was assessed using variance inflation factors (VIFs). All VIF values were below 5, indicating acceptable levels of multicollinearity and supporting the robustness of subsequent model estimation [50]. Mplus 8.3 was used for confirmatory factor analysis (CFA) and to test the mediating effect and path differences proposed in the research hypotheses. We validated the structural validity of all five scales used in this study using CFA. The results of the CFA indicated that the dimensional structure of the present study fit well with the original scales, suggesting good structural validity. Importantly, we further performed cross-validation and cross-group difference analysis of the model.

In addition to standard CFA and mediation analysis, we conducted split-sample cross-validation to evaluate the reproducibility and stability of the structural model. The full dataset was randomly divided into two equal subsamples, and the hypothesized model was tested independently in each. We compared key fit indices (e.g., CFI, RMSEA) and path coefficients across subsamples to assess consistency. This approach helps mitigate overfitting and has been recommended for enhancing model robustness in SEM applications [51].

## 3. Results

### 3.1. Descriptive Statistics

Table 1 shows the demographic information, mean, and standard deviation for the included participants’ sex, age, educational level, employment status, marital status, monthly income, interpersonal relationships, intensity and duration of square-dancing exercise, and group size.

### 3.2. Common Method Variance Test

To address potential common method variance (CMV) arising from the use of self-reported data, several procedural and statistical controls were implemented. During the survey design phase, anonymity was assured, item order was counterbalanced, and ambiguous or leading language was avoided to minimize acquiescence bias. For statistical control, Harman’s single-factor test was conducted prior to data analysis, applying unrotated principal component analysis (PCA) to all measurement items [52]. The first factor accounted for 36.83% of the total variance, which falls below the commonly accepted threshold of 40% [53], suggesting that CMV was not a major concern. Additionally, confirmatory factor analysis (CFA) was performed to further examine the CMV hypothesis. The single-factor model demonstrated poor fit (*χ*^2^/*df* = 73.355, RMSEA = 0.119, CFI = 0.475, TLI = 0.458), providing further evidence against substantial CMV. Taken together, these results indicate that CMV did not significantly affect the validity of the findings.

### 3.3. Related Analysis

The average, standard deviation, and Pearson’s correlation matrix of each variable are presented in Table 2. There were significant positive correlations between square-dancing exercise and cognitive reappraisal, prosocial behavior, and PsyCap (*r* = 0.040–0.835, *p*s < 0.01). Correlation analysis of each dimension of the scale revealed that, except for the resilience dimension of PsyCap, which was not significantly correlated (*p* > 0.05), square-dancing exercise was significantly and positively correlated with cognitive reappraisal (*r* = 0.132, *p*s < 0.01), prosocial behavior (*r* = 0.040–0.727, *p*s < 0.01), and PsyCap (*r* = 0.077–0.835, *p*s < 0.01). Cognitive reappraisal was significantly and positively correlated with prosocial behavior (*r* = 0.040–0.727, *p*s < 0.01), and cognitive reappraisal (*r* = 0.132–0.835, *p*s < 0.01) and PsyCap (*r* = 0.040–0.835, *p*s < 0.01) were significantly and positively correlated. The above results reveal that this approach is suitable for further mediating effect analysis.

### 3.4. Cross-Sequential Comparison

Next, we examined how trends in square-dancing exercise and PsyCap evolved after the pandemic (over almost 6 months). A cross-sequential comparison revealed a slight downward trend in the prevalence of square-dancing exercise (duration, intensity, and frequency) and PsyCap (self-efficacy, optimism, and hope), with t tests indicating statistical significance (*p* < 0.01), except for the resilience of PsyCap, which was not significantly correlated (*p* > 0.05); specific data are shown in Table 3.

### 3.5. Mediating Effect Analysis of Cognitive Reappraisal and Prosocial Behavior

**Figure 2:**
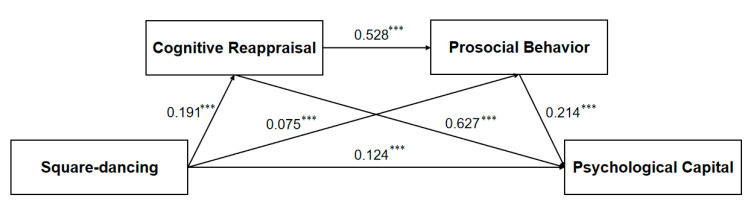
Hypothetical path model of this study (*N* = 4973). Notes: *** *p* < 0.001; the data are standardized path coefficients.

Prior to mediation analysis, we first established the baseline relationship between square dancing and psychological capital (PsyCap). The initial model demonstrated acceptable fit (*χ*^2^*/df* = 77.30, CFI = 0.93, TLI = 0.88, RMSEA = 0.12, SRMR = 0.04), revealing a significant positive direct effect (*β* = 0.283, *p* < 0.001). We then examined two single-mediator models: Model 1 incorporated cognitive reappraisal as a mediator, while Model 2 featured prosocial behavior. Both models achieved good fit (Model 1: *χ*^2^*/df* = 57.85, CFI = 0.94, TLI = 0.91, RMSEA = 0.11, SRMR = 0.03; Model 2: *χ*^2^*/df* = 31.12, CFI = 0.94, TLI = 0.92, RMSEA = 0.08, SRMR = 0.03). Bias-corrected bootstrap tests (5000 samples) confirmed significant mediation effects: cognitive reappraisal accounted for 50.2% of the total effect (effect = 2.404, 95% *CI* [1.893, 2.959]), while prosocial behavior mediated 33.7% (effect = 1.629, 95% *CI* [1.213, 2.080]). Finally, we tested a serial mediation model (Model 3), which exhibited excellent fit (*χ*^2^/*df* = 27.65, CFI = 0.94, TLI = 0.93, RMSEA = 0.07, SRMR = 0.03). All pathways in this model reached statistical significance, with standardized coefficients detailed in Figure 2.

Path analysis (Figure 2) revealed significant positive associations. Specifically, greater engagement in square dancing was associated with higher levels of PsyCap (*β* = 0.124, *p* < 0.001), enhanced cognitive reappraisal (*β* = 0.191, *p* < 0.001), and increased prosocial behavior (*β* = 0.075, *p* < 0.001) among middle-aged and older adults. Furthermore, both cognitive reappraisal (*β* = 0.627, *p* < 0.001) and prosocial behavior (*β* = 0.214, *p* < 0.001) were significant positive predictors of PsyCap. Cognitive reappraisal also significantly predicted greater prosocial behavior (*β* = 0.528, *p* < 0.001). Bias-corrected bootstrap analyses (5000 samples) confirmed significant mediating pathways. The indirect effect through cognitive reappraisal alone was 2.043 (95% *CI* [1.587, 2.529]), accounting for 42.5% of the total effect. The indirect effect solely through prosocial behavior was 0.274 (95% *CI* [0.131, 0.441]), explaining 5.7% of the effect. Critically, the serial indirect effect through both cognitive reappraisal and prosocial behavior was also significant (Effect = 0.369, 95% *CI* [0.275, 0.482]), representing 7.7% of the total effect. These results collectively demonstrate that cognitive reappraisal and prosocial behavior act as serial mediators in the relationship between square dancing participation and enhanced PsyCap. Detailed path coefficients are presented in Table 4.

Finally, we compared the three mediating paths and found that the mediating effect of path one (square-dancing—cognitive reappraisal—PsyCap) was significantly greater than that of path two (square-dancing—prosocial behavior—PsyCap) (95% *CI* [1.282, 2.266], *p* < 0.001) and path three (square-dancing—cognitive reappraisal—prosocial behavior—PsyCap) (95% *CI* [1.285, 2.111], *p* < 0.001). However, there was no significant difference between path two and path three (*p* > 0.05). Thus, cognitive reappraisal plays a more critical role in the relationship between square-dancing and PsyCap.

### 3.6. Cross-Validation

**Figure 3:**
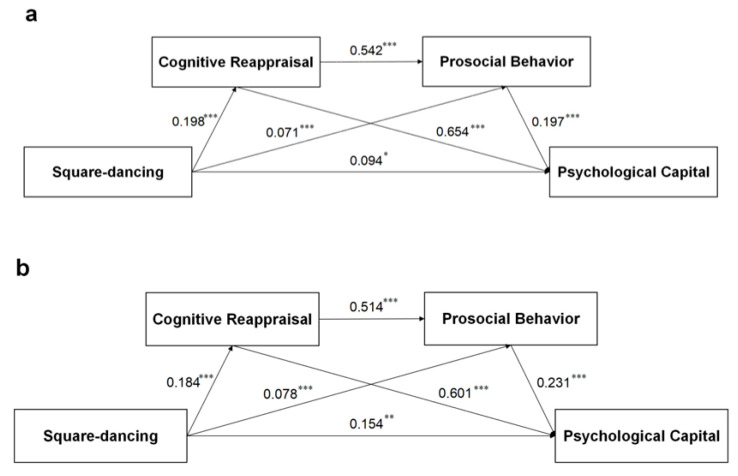
Cross-validation model. (**a**) Model for subsample 1; (**b**) Model of subsample 2. Notes: *** *p* < 0.001, ** *p* = 0.009, * *p* = 0.01; The data are standardized path coefficients.

To further demonstrate the stability of the serial mediation model constructed in this study and to increase the measurement reliability of the model, we adopted a cross-validation approach. Cross-validation can provide a reliable estimate of the generalizability of a model and evaluate its stability. In this study, we took the total sample (*n* = 4973), applied the RAND () function in Microsoft Excel 2016 to generate a sequence, and randomly divided it into two halves (*n*_1_ = 2487; *n*_2_ = 2486). Separate serial mediation models were constructed exactly as described in the previous section, and the fit indicators and path effects were measured. The model for the two random subsamples was again validated, and the model values are similar to those of the total sample. For additional details, see Table 5 and Figure 3. This cross-validation showed that the model was stable, which was consistent with the concept of split-half reliability.

### 3.7. Difference Tests of the Mediation Model

**Figure 4:**
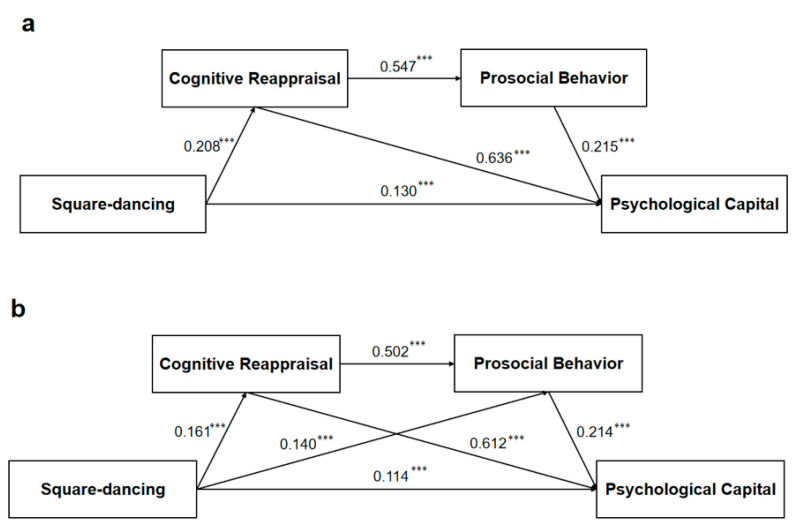
Research categorized model. (**a**) Model for middle-aged adults; (**b**) Model for older adults. Notes: *** *p* < 0.001; the data are standardized path coefficients.

Previous research has suggested that there are significant differences among age, monthly income level, and interpersonal relationships in terms of square-dancing, cognitive reappraisal, prosocial behavior, and PsyCap; therefore, this study examined the consistency of these mediating effects, and the model fit results for each categorical variable are shown in Table 6. First, the mediation models for middle-aged adults (*n*_a_ = 3054) and older adults (*n*_b_ = 1919) were examined separately. The fit indices of the two models are good (see Table 5 for details), and specific contents are shown in Figure 4. In Model b, all path coefficients were significant, and the standardized values of each coefficient are shown in Table 6. However, intuitively, the mediating effect of prosocial behavior is not significant in Model a (*p* > 0.05).

**Figure 5:**
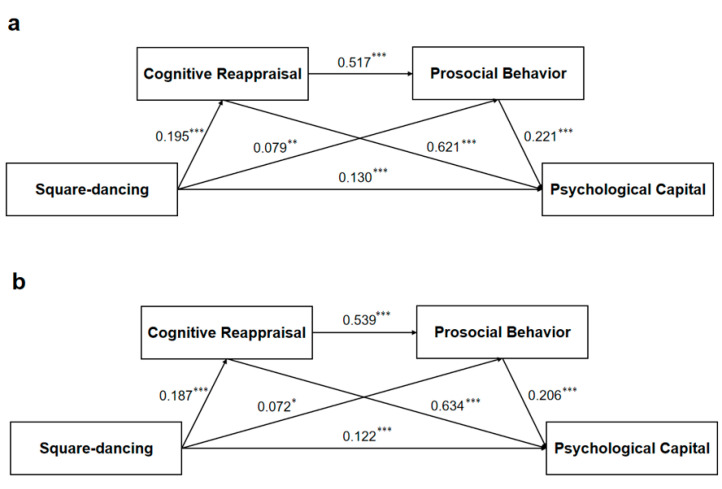
Research-categorized model. (**a**) Model for high-income; (**b**) Model for low-income. Notes: * *p* = 0.014, ** *p* = 0.007, *** *p* < 0.001; the data are standardized path coefficients.

Second, the mediation models of high income (*n*_a_ = 2430) and low income (*n*_b_ = 2543) were examined separately. The fit indices of the two models are good (see Table 4 for details). However, we did not find a difference between the two models across path analyses. The specific contents are shown in Figure 5. That is, there was no significant difference between high-income and low-income populations in terms of the relationship between square-dancing and PsyCap.

**Figure 6:**
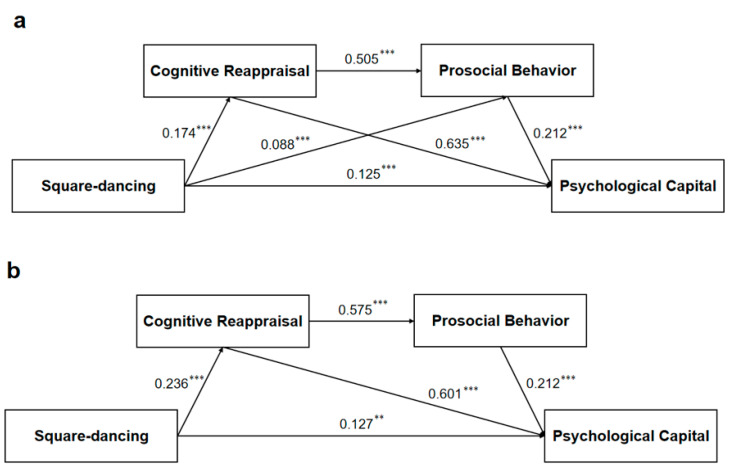
Research-categorized model. (**a**) Model for no distress; (**b**) Model for distress. Notes: ** *p* = 0.001, *** *p* < 0.001; the data are standardized path coefficients.

Finally, we categorized the continuous variable of interpersonal relationships into a no-distress group (*n*_a_ = 3994) and a distress group (*n*_b_ = 979). The index results also showed a good fit in Figure 6. In Model a, all path coefficients were significant, and the standardized values of each coefficient are shown in Table 6. However, the mediating effect of prosocial behavior is not significant in Model b (*p* > 0.05).

In summary, we investigated cross-group differences in age, monthly income, and interpersonal relationships. The mediating role of prosocial behavior alone did not hold for middle-aged adults or those in the interpersonal distress group. In contrast, this result was not observed in the high- and low-income subgroups.

## 4. Discussion

To our knowledge, this is the largest cross-sectional study of square dancing in China. The primary aim of this study was to explore the relationship between square-dancing exercise and PsyCap, and to uncover the underlying mechanisms from a positive psychology perspective by integrating a cross-sequential design and cross-validation. The research objective was effectively addressed through the present study. The findings confirmed a positive association between square-dancing and PsyCap and further identified emotion regulation strategies and prosocial tendencies as key mediators. The integration of cross-sectional and cross-sequential approaches strengthened the temporal validity of the conclusions.

Specifically, our key findings can be summarized as follows. First, the results supported the positive association between square-dancing exercise and PsyCap, and H1 was verified. Second, a serial mediation model was finalized on the premise that all individual mediators were valid, and H2, H3, and H4 were validated. Third, we conducted a cross-group difference test, which resulted in the partial validation of H5. Finally, cross-sequential comparison supported trend changes in PsyCap within 6 months, and the robustness of PsyCap was further demonstrated with a larger sample size.

### 4.1. Direct Relationship Between Square-Dancing Exercise and PsyCap

Selective Optimization with Compensation (SOC) suggests that although individuals experience the loss of various resources (e.g., physical and mental illnesses, social resources) during aging, they also encounter various opportunities, with potential growth and plasticity [54]. This study revealed that square-dancing exercise can positively predict PsyCap, i.e., middle-aged and older adults can be motivated to exercise, their positive emotional state can be maintained, their self-efficacy can increase, their optimistic attributions and hopefulness can improve, their social and emotional needs can be satisfied, and their PsyCap can increase. However, we found that square-dancing exercise was not associated with the resilience dimension of PsyCap, which contrasts with previous findings of resilience by PA [55]. With age and social experience, older adults may possess relatively high levels of resilience. Low- and moderate-intensity PA requires fewer difficulties and obstacles (e.g., helplessness, exhaustion) to overcome [56]. Moreover, much of the previous research on PA for resilience has focused on children and youth [57], but there is a gap in the empirical research on middle-aged and older adults, which is urgently needed [58]. Compared with the secondary data [40], there was a slight decrease in the PA and PsyCap. In the Chinese post-pandemic era, the physiological functions of middle-aged and older adults are in a state of recovery, individual healthy habits have changed, and due to physiological limitations, the frequency and duration of participation in square-dancing exercise have decreased. The motivation and adherence to participate in square-dancing exercise have inevitably changed.

### 4.2. Mediating Role of Cognitive Reappraisal and Prosocial Behavior Tendencies

The present study revealed that cognitive reappraisal mediates the relationship between square-dancing exercise and PsyCap, which is consistent with evidence from previous studies on the positive effects of PA on cognitive reappraisal [59] and cognitive reappraisal, which favors PsyCap [60]. Square-dancing exercise enhances PsyCap through a dual-path mechanism mediated by cognitive reappraisal and prosocial behaviors. The dynamic music rhythm and group-based format of square dancing create a positive psychological experience that reduces negative emotions while enhancing cognitive reappraisal and self-determination (autonomy and sense of control) [61]. These immediate psychological improvements directly increase subjective well-being and lay the foundation for PsyCap development. Concurrently, the inherent social nature of group dancing fosters increased willingness for interpersonal interaction. Through sustained communication and coordinated movements, participants develop prosocial behavior tendencies [62]. On the basis of self-determination theory (SDT), this behavioral shift is further reinforced by the emotional benefits of exercise: individuals with improved affective states are more motivated to engage socially, thereby amplifying prosocial behaviors and strengthening social competence [63].

Furthermore, we found that the mediating effect of cognitive reappraisal alone had the largest effect size among the paths. According to cognitive behavioral theory (CBT), behaviors are elicited by emotions and reinforced by cognition [64]. Therefore, middle-aged and older adults who have participated in square dancing for a long period may have deeper knowledge and understanding of their self-emotions, and prosocial behavior tendencies usually occur after an individual’s positive thinking and emotions are improved [65]. Second, although the serial mediation effect in this study was not as large as the separate mediation effect of cognitive reappraisal, it was still statistically significant, suggesting that the joint roles played by cognitive reappraisal and prosocial behavior cannot be ignored. Finally, we also used cross-validation to further verify the stability and reliability of the model [66]. The results show that the models of the two random samples fit well, and the coefficients of each path are consistent with those of the total sample model, which again validates the conclusions.

### 4.3. Cross-Group Analysis of the Serial Mediation Model

From a demographic point of view, first, this study revealed that the mediating role of prosocial behavior was not significant in the middle-aged group (45–65 years old) after the age difference was tested. According to the theory of social-emotional selectivity, prosocial attitudes and behaviors change with age and experience [67]. Therefore, older adults attach more importance to social emotions and show greater empathy toward others, thus investing more in social interactions, displaying well-intentioned behaviors, and increasing the accumulation of positive emotions [68]. In addition, there was no significant difference between the two models (high/low monthly income groups), meaning that the relationship between square-dancing exercise and PsyCap was consistent for the two groups. While income may be an important influence on PsyCap [15], some studies in the Chinese context have reported no significant role of income in promoting the development of PsyCap [40]. However, this result needs to be further explored and confirmed.

This study also focused on the level of interpersonal relationships from a social interaction perspective and revealed that the mediating effect of prosocial behavior tendency in groups with interpersonal relationship distress was not significant. Individuals are afraid of engaging in social interactions and participating in interpersonal interactions, which makes it more difficult to generate behaviors such as obedience and altruism, making them less likely to exhibit prosocial behavior tendencies [69].

### 4.4. Limitations and Future Directions

The present study has several limitations. First, the significant gender imbalance (95.7% female participants) reflects square dancing’s inherent social dynamics, where female dominance aligns with traditional gender roles (e.g., women as community coordinators) and activity traits (e.g., rhythmic, noncompetitive choreography) [70]. While this imbalance may be due to program characteristics and trends in square dancing rather than sampling bias [71], it limits the generalizability of the findings to middle-aged and older men and to broader populations. Second, methodological and contextual factors may affect the robustness of the findings. The use of self-reported data introduces potential biases (e.g., social desirability, recall, and common method bias), and the cross-sectional design limits causal inference. Additionally, the questionnaire was not specifically tailored for older adults, particularly regarding prosocial behavior. Third, the cultural specificity of square dancing in China may restrict the applicability of the findings to other cultural or demographic contexts where this activity is less prevalent or culturally significant.

To address these limitations, future studies should adopt a mixed-methods design that integrates experimental and follow-up studies to elucidate the causal mechanisms among variables. Additionally, efforts should be made to diversify the participant pool (e.g., including more male participants) and explore culturally adaptive forms of square dancing to enhance generalizability and theoretical robustness.

### 4.5. Implications

The present study, which is based on positive psychology and active aging, focuses on the effect of square dancing on the positive psychological qualities of middle-aged and older adults and has important theoretical and practical significance. At the methodological level, we have increased the depth of the study; SEM was used to explore the serial mediation effect; cross-validation was conducted to examine the stability of the model; subgroup analyses clarified the roles of age, monthly income, and interpersonal relationships; and cross-sequential comparison explored the developmental process of PA and PsyCap, enriching the diversity and depth of the present study.

## 5. Conclusions

In the context of active aging, the present study measured the experiences of more than 5000 Chinese middle-aged and older adults in square dancing. We established a reliable and generalizable SEM using multiple methods and identified serial-mediated relationships among square-dancing exercise, PsyCap, cognitive reappraisal, and prosocial behavior. Moreover, on the basis of a large sample of data, we verified the stability and generalizability of the model by cross-sectioning halves, indicating reproducibility. In addition, the SEM indicated cross-group differences in both age and interpersonal relationships, but not for high or low income. Future research should explore the intrinsic mechanisms and other potential factors among variables and introduce experimental studies to explore causality.

## Figures and Tables

**Table 1:** Demographic information of the participants (*N =* 4973).

Sociodemographic Characteristics	*M* (*SD*) or *N* (%)
**Age (years)**	59.14 (7.17)
**Sex**	
Female	4760 (95.7%)
Male	213 (4.3%)
**Highest level of education**	
Primary school	306 (6.2%)
Junior middle school	1765 (35.5%)
Senior middle school	1887 (37.9%)
Bachelor’s degree or above	1015 (20.4%)
**Employment status**	
Employed	872 (17.5%)
Retired	4101 (82.5%)
**Marital status**	
Married	4726 (95.0%)
Unmarried	247 (5.0%)
**Monthly income**	
RMB 0–3500	2543 (51.2%)
RMB 3500 or more	2430 (48.8%)
**Square dance intensity**	17.42 (13.24)
**Square dance group size**	
<25 people	2797 (56.0%)
>25 people	2176 (44.0%)
**Square-dancing exercise duration**	
<5 years	2021 (40.9%)
>5 years	2952 (59.1%)
**Interpersonal relationships**	
No Distress	3994 (80.3%)
Distress	979 (19.7%)

Notes: *M* = mean; *SD* = standard deviation; *N* = absolute frequency; % = relative frequency.

**Table 2:** Descriptive statistics and correlation matrix of each variable (*N* = 4973).

Variables	*M*	*SD*	1	2	3	4	5	6	7	8	9	10	11
1. PARS-3	17.42	13.24	_										
2. ERQ-CR	33.73	5.70	0.132 **	_									
3. PTM-Public	12.40	5.01	0.040 **	0.239 **	_								
4. PTM-Obedient	20.58	4.34	0.124 **	0.421 **	0.345 **	_							
5. PTM-Urgent	12.19	2.57	0.080 **	0.442 **	0.305 **	0.707 **	_						
6. PTM-Emotional	19.61	4.36	0.093 **	0.465 **	0.377 **	0.683 **	0.727 **	_					
7. PTM-Altruistic	16.67	3.94	0.060 **	0.366 **	0.123 **	0.494 **	0.552 **	0.511 **	_				
8. PTM-An	19.95	4.45	0.077 **	0.437 **	0.242 **	0.557 **	0.630 **	0.617 **	0.607 **	_			
9. PPQ-SE	37.77	7.47	0.196 **	0.642 **	0.255 **	0.403 **	0.418 **	0.429 **	0.317 **	0.377 **	_		
10. PPQ-Resilience	30.34	6.11	0.007	0.209 **	0.162 **	0.161 **	0.147 **	0.161 **	0.077 **	0.121 **	0.319 **	_	
11. PPQ-Hope	33.46	5.72	0.165 **	0.671 **	0.166 **	0.387 **	0.420 **	0.405 **	0.355 **	0.381 **	0.757 **	0.084 **	_
12. PPQ-Optimism	34.90	5.65	0.157 **	0.719 **	0.231 **	0.436 **	0.452 **	0.445 **	0.358 **	0.421 **	0.757 **	0.260 **	0.835 **

Notes: ** *p* < 0.01; Abbreviations: CR, Cognitive reappraisal; SE, Self-efficacy; An, Anonymous.

**Table 3:** Independent samples *t*-test for square-dancing exercise and PsyCap.

Variables	Secondary Data *M* (*SD*)	Main Data *M* (*SD*)	*t*
**Square-dancing exercise**	21.55 (14.42)	17.42 (13.24)	11.69 **
Duration	2.75 (1.18)	2.08 (0.93)	26.57 **
Intensity	1.86 (0.68)	2.04 (0.71)	−10.57 **
Frequency	4.05 (0.93)	3.75 (1.17)	10.93 **
**PsyCap**	139.85 (19.64)	136.46 (19.84)	6.92 **
PPQ-Self-efficacy	39.25 (7.55)	37.77 (7.47)	8.00 **
PPQ-Resilience	30.44 (6.3)	30.34 (6.1)	0.70
PPQ-Hope	34.28 (5.80)	34.90 (5.65)	5.77 **
PPQ-Optimism	35.87 (5.44)	33.46 (5.72)	7.05 **
*M* (*SD*), Range			
Age (years)	60.62 (6.68), 45–85	59.14 (7.17), 45–85	

Notes: ** *p* < 0.01. Abbreviations: *M* = mean; *SD* = standard deviation; Secondary data (March to April 2023); Main data (August to September 2023).

**Table 4:** Bias-corrected bootstrap estimates for model path coefficients.

Intermediary Process	Effect Type	Effect Value	Bootstrapped LLCI ^a^	Bootstrapped ULCI ^a^	Effect Size (%)
Square-dancing—cognitive reappraisal—psycap	Mediating effect	2.404	1.893	2.959	50.2
Square-dancing—prosocial behavior tendency—psycap	Mediating effect	1.629	1.213	2.080	33.7
Square-dancing—cognitive reappraisal—prosocial behavior tendency—psycap	Serial mediating effect	0.369	0.275	0.482	7.7
	Mediating effect of CR	2.043	1.587	2.529	42.5
	Mediating effect of PB	0.274	0.131	0.441	5.7
Total effect		4.804	4.046	5.615	

Notes: ^a^ Boot LLCI and Boot ULCI refer to the 95% confidence of the indirect effect estimated by the bias-corrected bootstrap method, respectively, the upper and lower limits of intervals, respectively. CI, Confidence intervals; CR, cognitive reappraisal; PB, prosocial behavior tendency.

**Table 5:** Fit indexes of the cross-validation and total models.

Categorization	Group Size (*n*)	χ^2^/*df*	CFI	TLI	RMSEA	SRMR
Subsample 1	2487	13.29	0.95	0.94	0.07	0.03
Subsample 2	2486	15.26	0.94	0.92	0.08	0.03
Total sample	4973	27.65	0.94	0.93	0.07	0.03

**Table 6:** Fit indexes of the categorized models.

Categorization	Levels	Group Size (n)	χ^2^/*df*	CFI	TLI	RMSEA	SRMR
Age	Middle-age	3054	16.38	0.95	0.93	0.07	0.03
	Older	1919	12.43	0.94	0.92	0.08	0.03
Income	High	2430	13.30	0.95	0.93	0.07	0.03
	Low	2543	15.02	0.94	0.93	0.07	0.03
Interpersonal relationships	No distress	3994	21.91	0.94	0.93	0.07	0.03
	distress	979	6.61	0.95	0.93	0.08	0.04

## Data Availability

The authors acknowledge that all data generated or analyzed during this study are included in this published article. The datasets presented in this article are not readily available because they are part of an ongoing sub-study. Requests to access the datasets should be directed to the corresponding author.

## Abbreviations

The following abbreviations are used in this manuscript:

PA	Physical activity
PsyCap	Psychological capital
